# Malignant peripheral nerve sheath tumor in early childhood: a case report of a diagnostic challenge

**DOI:** 10.3389/fonc.2025.1609477

**Published:** 2025-06-24

**Authors:** Xin Zhang, Wenbin Guan, Jianqing Liang

**Affiliations:** ^1^ Department of Pediatric Hematology/Oncology, Xinhua Hospital Affiliated to Shanghai JiaoTong University School of Medicine, Shanghai, China; ^2^ Department of Pathology, Xinhua Hospital Affiliated to Shanghai JiaoTong University School of Medicine, Shanghai, China; ^3^ State Key Laboratory of Organ Failure Research, National Clinical Research Center of Kidney Disease, Division of Nephrology, Nanfang Hospital, Southern Medical University, Guangzhou, China

**Keywords:** malignant peripheral nerve sheath tumor, neurofibromatosis type 1, atypical neurofibroma, plexiform neurofibroma, CDKN2A

## Abstract

**Background:**

Malignant peripheral nerve sheath tumors (MPNST) are a severe complication of neurofibromatosis type 1 (NF1). The median age at diagnosis is 47.1 years in the general population, whereas NF1-associated cases present significantly earlier, at 33.9 years. The incidence is 1.46 per million in the general population and 0.56 per million in children, with a higher occurrence in late adolescence.

**Case report:**

A 3-year-old girl was referred for a recurrent soft-tissue mass in her left wrist. Imaging suggested a neurofibroma. After excision, pathological analysis and genetic testing revealed a germline *NF1* mutation, confirming Neurofibromatosis type 1 (NF1), along with somatic mutations in *NF1* and deletions in *CDKN2A*. Pathology confirmed malignant Triton tumor (MTT). She received six cycles of ifosfamide and doxorubicin, followed by a second excision, which showed persistent tumor activity. Positron emission tomography–computed tomography (PET-CT) scans revealed reduced metabolic activity at the tumor site, suggesting a partial response to treatment. The patient is currently undergoing oral MEK inhibitor-targeted therapy.

**Conclusion:**

This case highlights the challenges associated with MPNST in pediatric NF1. Both surgery and chemotherapy have been shown to enhance outcomes. Ongoing monitoring is crucial, and additional research on MEK inhibitors and genetic profiling is imperative for tailoring treatment strategies.

## 1 Introduction

Neurofibromatosis type 1 (NF1) is an autosomal dominant disorder caused by mutations in *NF1*, with a global incidence of approximately 1 in 3,000 live births (1, 2). Among NF1 patients, 30%–60% develop plexiform neurofibromas (PN), which typically manifest prenatally or during early childhood. Although classified as benign neoplasms, 8%–13% of PN undergo malignant transformation into malignant peripheral nerve sheath tumors (MPNST). Notably, MPNST is exceptionally rare in children under 10 years of age, with its incidence increasing sharply during adolescence. In pediatric and young adult populations (0–19 years), approximately 50% of MPNST cases arise in the context of NF1 (associated with biallelic NF1 inactivation), 40% occur sporadically, and 10% are radiation-induced (3). The overall annual incidence of MPNST is 1.46 per million population, but pediatric rates are markedly lower: 0.56 per million in children under 15 years of age, with the majority of cases clustered in adolescents aged 10–19 years (4, 5).

## 2 Case presentation

**Figure 1:**
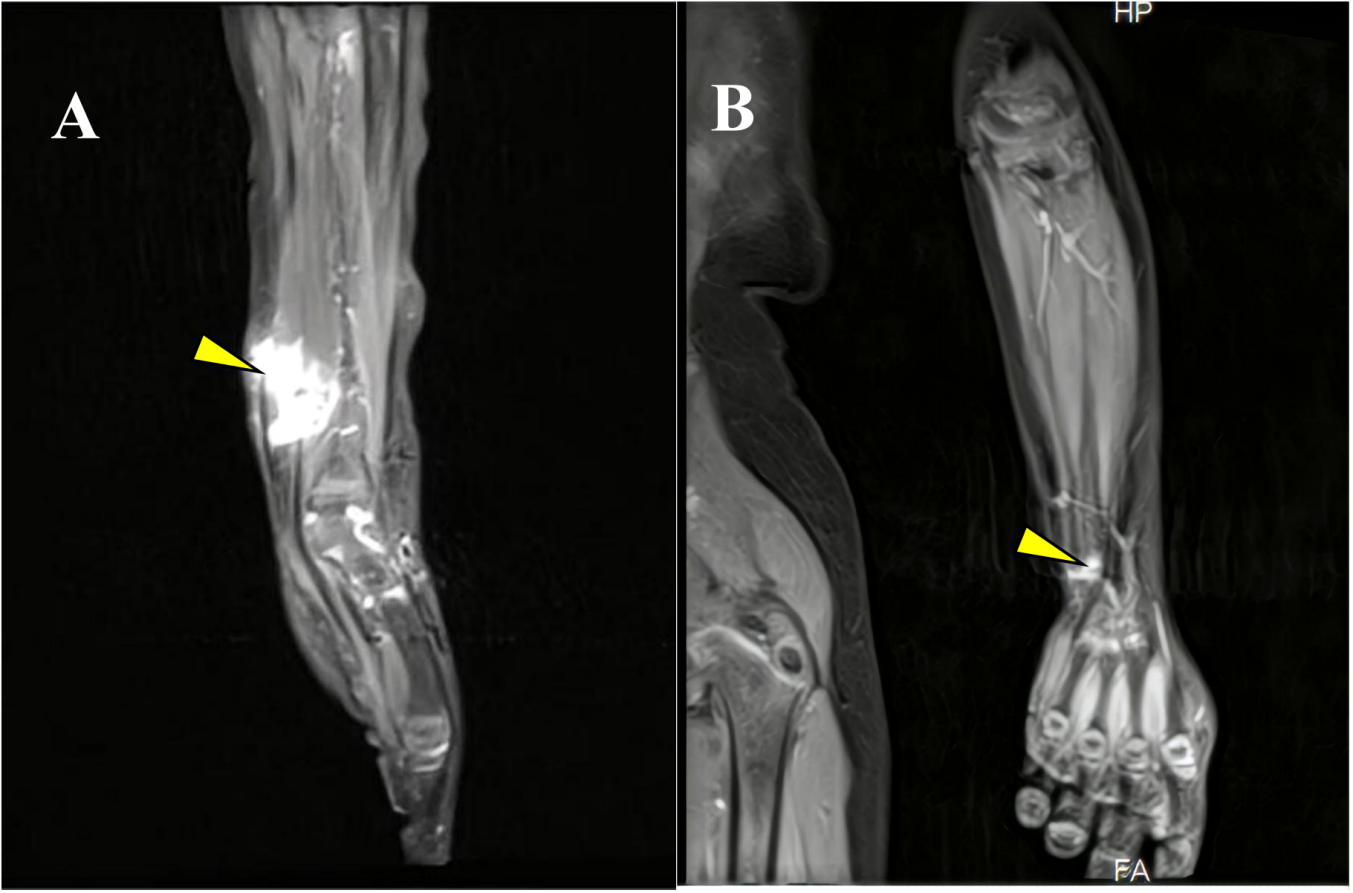
**(A)** MRI highlighting the tumor in the left wrist at initial presentation. **(B)** Follow-up MRI demonstrating the post-treatment status of the left wrist lesion (yellow arrow).

**Figure 2:**
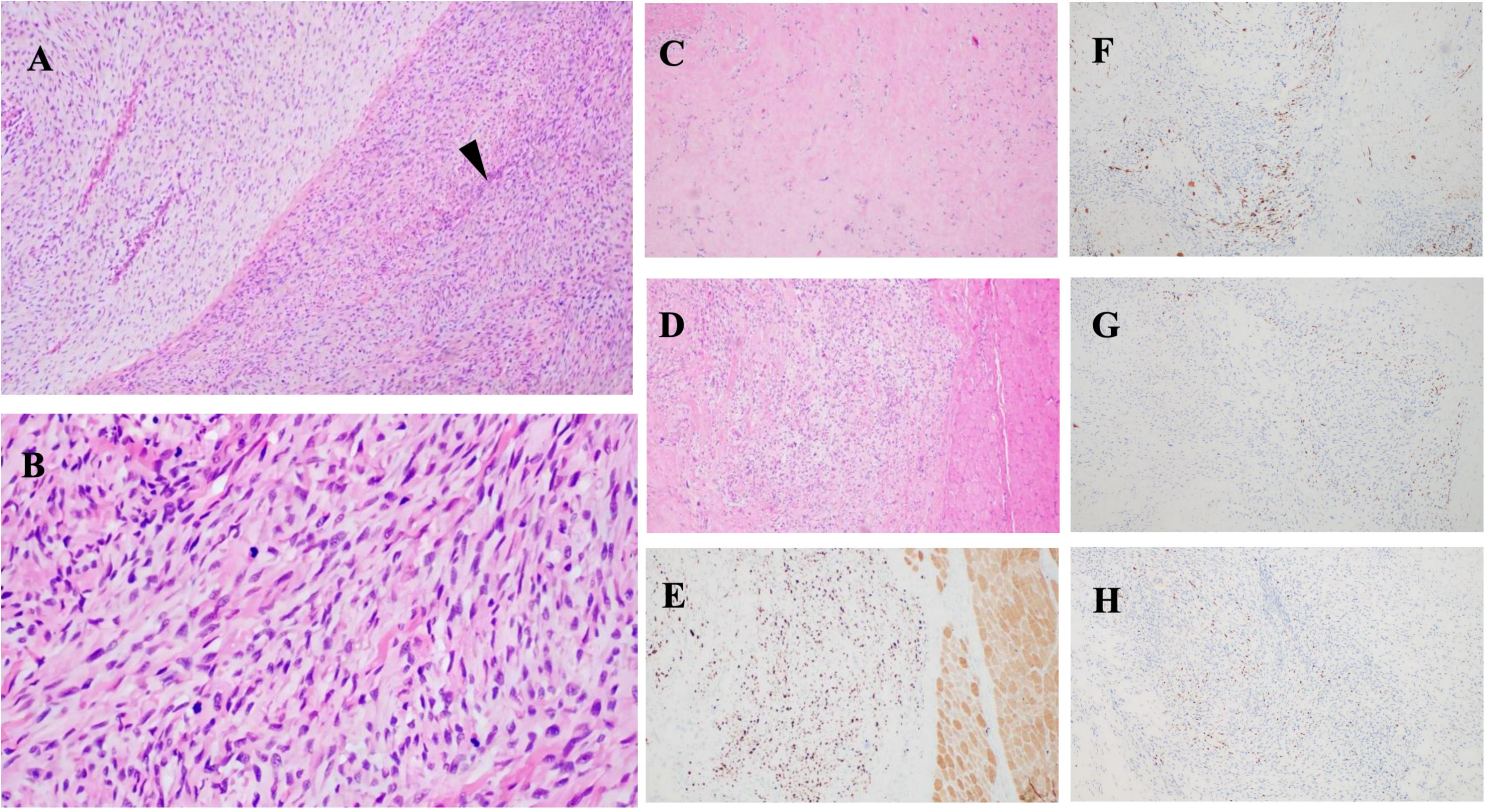
Histopathological images: **(A, B)** Tumor features before treatment. H&E staining shows malignant peripheral nerve sheath tumor (MPNST) characteristics, including “fish-bone” pattern and spindle-shaped cells associated with peripheral nerves (black arrowheads). **(A)** ×100; **(B)** ×400. **(C, D)** Post-treatment images show hyaline degeneration and residual tumor areas. **(E)** Ki67 immunohistochemistry indicates cellular proliferation. Tumor cells are positive for **(F)** desmin, **(G)** myogenin (MyoG), and **(H)** MyoDI, confirming differentiation.

A 3-year-old girl initially presented with a left wrist mass in September 2023, identified as a cyst, on ultrasound at an external hospital. The family opted for surgical removal of the lesion On November 12, 2023; however, the initial pathological diagnosis was inconclusive. Subsequently, the patient was referred to our pediatric oncology department for further evaluation and definitive diagnosis. Upon detailed history and physical examination, the patient was asymptomatic, showing no pain or skin lesions. Physical examination revealed multiple café-au-lait macules with no other abnormalities. Her medical history was devoid of prior malignancies, recent systemic symptoms, trauma, or family history of pediatric cancers. Imaging studies were conducted. Ultrasound revealed a 2×2 cm cystic lesion in the left forearm. On March 4, 2024, Magnetic resonance imaging (MRI) showed a healthy alignment of the left wrist joint. However, an abnormal signal lesion was observed within the soft tissue interval adjacent to the distal radius. Specifically, this lesion covered an area of approximately 1.7 × 1.6 × 2.4 cm on the dorsal aspect of the left forearm, consistent with a neurofibroma (**Figure 1A**). Considering the lesion location and imaging features, the differential diagnoses included plexiform neurofibromas, lymphangiomas, and rhabdomyosarcomas. Cranial MRI did not reveal any abnormalities elsewhere, supporting the diagnosis of NF1. The clinical picture raised suspicion of NF1, but a diagnosis based solely on the NIH 1987 consensus clinical criteria remains uncertain. To clarify this, we recommended peripheral blood genetic testing and convened a multidisciplinary team discussion. On March 21, 2024, given the progressive enlargement of the tumor, surgical excision was advised in pediatric surgery for a definitive histopathological diagnosis. On March 28, 2024, pathological examination revealed tumor cells negative for P16, SOX10, S100, and H3K27me3, indicating potential malignancy (**Figures 2A, B**). The margins were clear after the first surgery, and the patient was enrolled in the follow-up protocol. Genetic testing during this period revealed the presence of germline and somatic *NF1* mutations. On May 28, 2024, a follow-up MRI of the forearm revealed post-surgical changes at the site of the spindle cell tumor. Additionally, abnormal signals around the distal radius of the left forearm raised suspicion of recurrence. Based on the gene testing results and imaging studies indicating tumor recurrence, we re-evaluated the pathological features of the tumor tissue obtained during the March 21, 2024, surgery to establish the final diagnosis. Subsequent pathological examination revealed tumor cells with positive immunoreactivity for desmin, myogenin (MyoG), and MyoD1, with a Ki-67 proliferation index that exceeded 60%. The tumor tested negative for S100, SOX10, H3K27me3, and other markers associated with benign nerve sheath tumors. Genomic profiling revealed an NF1 heterozygous mutation (NM_000267:c.7267dupA, p.T2423fs), deletions in *CDKN2A*, and amplification of *MDM2* and *CDK4*.

Histopathological examination revealed a tumor composed of numerous highly atypical spindle cells accompanied by focal necrosis, indicating malignancy. Immunohistochemical analysis demonstrated positivity for desmin, MyoG, and MyoD1 (**Figures 2F–H**), confirming the presence of heterologous rhabdomyoblastic differentiation. Notably, S100 protein expression was persistently negative in this case, suggesting tumor dedifferentiation, epigenetic dysregulation, and malignant transformation. The absence of S100 aligns with the genetic alterations typically observed in NF1-associated MPNST, particularly in tumors exhibiting heterologous differentiation that often lack Schwannian markers. Molecular studies revealed loss of p16 and H3K27me3 expression, epigenetic modifications strongly associated with NF1-related MPNST. Additionally, germline and somatic *NF1* mutations, along with *CDKN2A* deletion, further support this diagnosis. Given the patient’s confirmed diagnosis of NF1, the tumor is classified as a malignant peripheral nerve sheath tumor with rhabdomyoblastic differentiation—specifically, a malignant Triton tumor (MTT) (6, 7).

**Figure 3:**
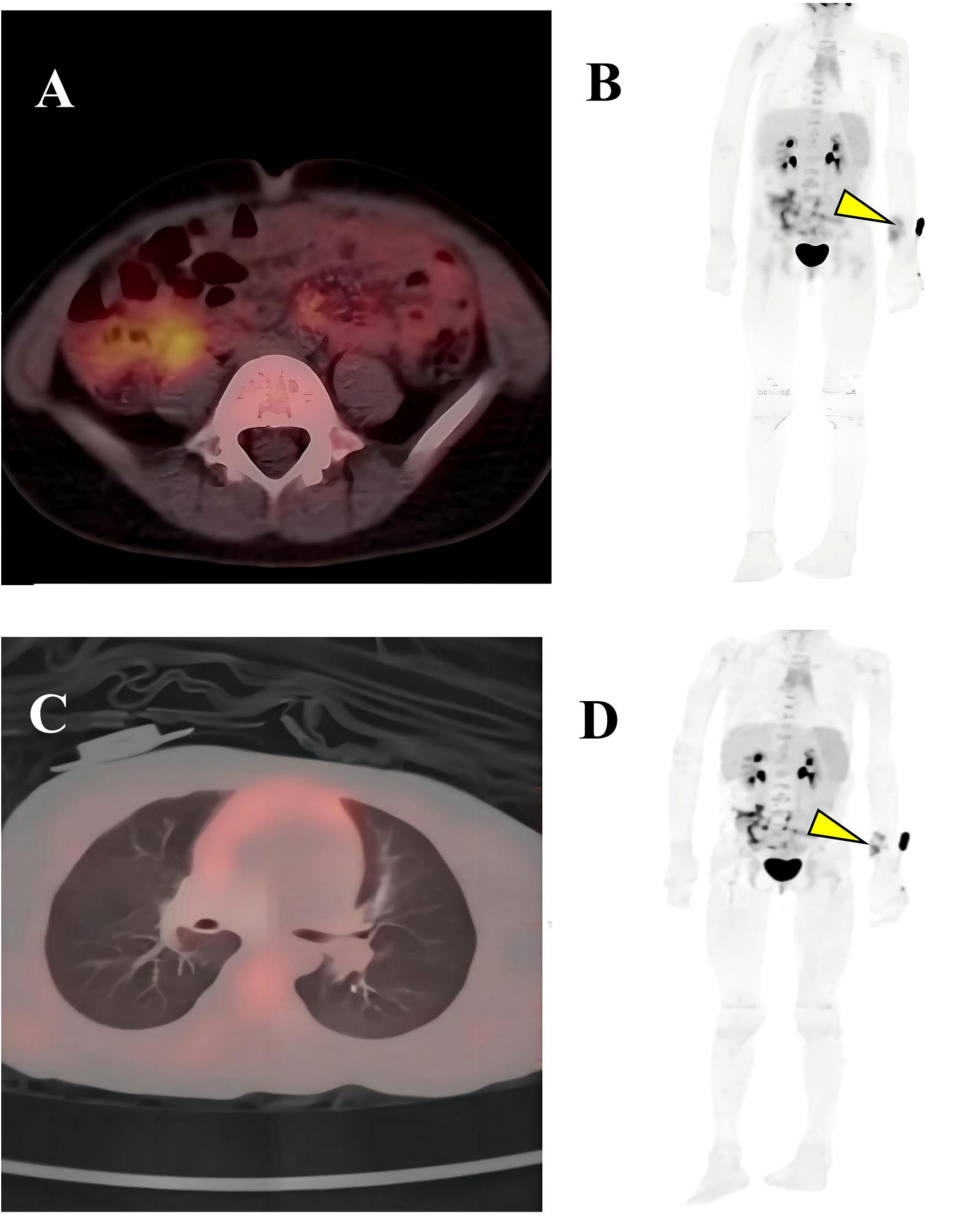
PET-CT imaging. **(A, B)** Showed soft tissue changes with increased FDG uptake; **(C, D)** Slightly elevated FDG uptake suggests residual tumor, but no systemic metastases are observed. Residual lesion in the left wrist (yellow arrow).

**Figure 4:**
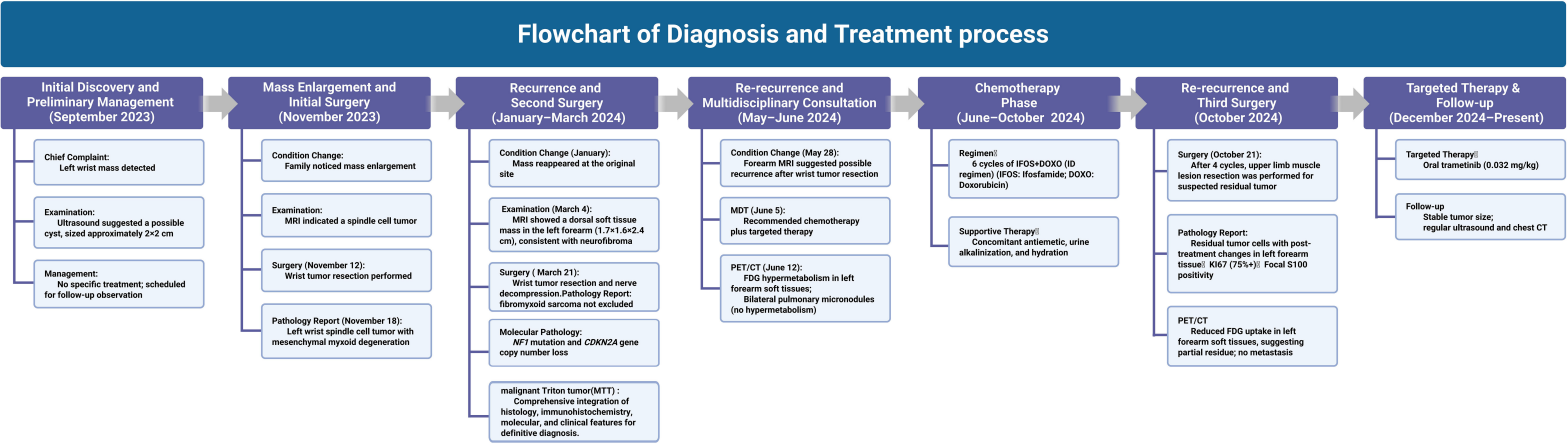
Overview of the therapeutic interventions and efficacy evaluations for this case along the timeline.

On June 5, 2024, the multidisciplinary team (MDT) consultation advised hospitalization for chemotherapy combined with targeted therapy.By June 12, 2024, the positron emission tomography–computed tomography (PET-CT) scan revealed soft tissue changes accompanied by increased FDG uptake, with no evidence of metastasis (**Figures 3A, B**). On June 19, 2024, the patient underwent six cycles of chemotherapy with ifosfamide and doxorubicin (ID regimen), supplemented with antiemetics, alkalization, and hydration therapy. After completing four cycles, the patient was scheduled for surgical resection of the residual tumor tissue under general anesthesia on October 24, 2024. A pathological review of the resected tissue revealed residual tumor cells with immunohistochemical profiles consistent with malignant rhabdoid features. Briefly, the post-treatment pathology and immunohistochemistry profiles were as follows: KI67 (75%+), focal positivity for S100, negative for SOX10, H3K27Me3, SMA, CD34, β-catenin, ALK and MTAP. Desmin, MyoG, and MyoD1 all exhibited scattered positivity (**Figures 2C–E**). Follow-up PET-CT scans on November 12, 2024 (**Figures 3C, D**), revealed postoperative changes in the left wrist, with slight FDG uptake in the soft tissues around the mid-to-distal radius, suggestive of residual tumor tissue, but no evidence of distant metastasis, when compared with previous imaging from June 13, 2024. The patient’s clinical condition improved significantly, with no further tumor growth observed. Follow-up MRI shows persistent abnormal signals in the soft tissue of the lower left forearm (**Figure 1B**). Since December 2024, she has been maintained on oral trametinaib at a dose of 0.032 mg/kg. Her clinical condition remained stable, with notable symptom relief. Routine follow-up includes ultrasound examinations to monitor tumor recurrence and periodic chest CT scans to survey possible lung metastases. This case illustrates the importance of integrating imaging, histopathology, and molecular genetics in diagnosing and managing complex soft tissue tumors associated with NF1. Through multidisciplinary cooperation, targeted therapies, and meticulous follow-up, we aimed to optimize the outcomes of these high-risk therapeutic interventions and the corresponding assessments of effectiveness are chronologically outlined in the timeline in **Figure 4**.

## 3 Discussion

Recent advancements in diagnosing and molecularly characterizing neurofibroma-associated tumors in patients with NF1 have significantly enhanced our understanding of tumor progression and classification. Neurofibromas (PN) in NF1 patients can progress into atypical neurofibromas (ANF), classified by the World Health Organization (WHO) as Atypical Neurofibroma of Uncertain Biological Potential (ANNUBP) (8). Studies have shown that post-activation of *CDKN2A* and ANF/ANNUBP can evolve into MPNST through mechanisms such as promoter methylation, gene deletions, or point mutations (8). MPNST exhibit notable histological heterogeneity, often overlapping with benign tumors; this outcome, which blurs the benign-malignant boundary, complicates diagnosis. The absence of specific immunohistochemical markers further hinders timely diagnosis. Clinically, the prognosis varies significantly. For instance, the five-year survival rate of the spindle cell subtype of MPNST is about 14%, with a median survival time of 13 months, a local recurrence rate of 50%, and a median time to recurrence or progression of only six months (9, 10). Tumor grade has been recognized as an independent prognostic factor influencing disease-specific survival (9, 10).

Asymptomatic ANF is typically managed through conservative observation, while rapidly growing or recurrent tumors often necessitate surgical intervention. Currently, no randomized studies have assessed the use of neoadjuvant or adjuvant chemotherapy for MPNST, and its role remains controversial. Guidelines for soft tissue sarcomas and retrospective analyses by the European Organisation for Research and Treatment of Cancer(EORTC) suggest that the prognosis of MPNST is similar to that of other soft tissue sarcomas. In the ISG-STS 1001 trial (11), 287 high-risk soft tissue sarcoma patients, including 27 with MPNST, were randomized (1:1) to receive either three cycles of standard chemotherapy (doxorubicin plus ifosfamide) or histology-tailored chemotherapy. MPNST patients received etoposide plus ifosfamide, with each cycle lasting 21 days. At 46 months of follow-up, the standard chemotherapy group showed significantly higher disease-free survival (62%) than the tailored chemotherapy group (38%) (P=0.004; HR=2.00), and the overall survival rates were 89% versus 64% (P=0.033; HR=2.687). Exploratory subgroup analysis indicated a hazard ratio of 2.38 for MPNST patients with MPNSTs, although this was not statistically significant, suggesting a potential continued benefit. Therefore, the combination of anthracyclines with ifosfamide based on histology remains the current standard for neoadjuvant chemotherapy in high-risk soft tissue sarcomas. For unresectable MPNSTs, radiotherapy may be considered, although its effect on long-term survival remains limited. Adjuvant chemotherapy can reduce mortality in residual or metastatic disease but does not prevent progression, underscoring the lack of standardized treatment protocols currently available (9, 10). Recent clinical trials have shown promising results with MEK inhibitors, such as selumetinib and mirdametinib, in NF1-related neurofibromas, demonstrating significant tumor shrinkage, pain relief, and improved quality of life with tolerable safety profiles (12, 13). In contrast, targeted therapies for MPNST remain challenging. Early phase II trials combining MEK and mTOR inhibitors have yielded encouraging responses, suggesting that multi-targeted approaches might overcome current therapeutic barriers (14).

Mechanistically, *CDKN2A* is a crucial tumor suppressor gene that encodes p16(INK4a) to inhibit the CDK4/6-RB pathway and p14(ARF) to stabilize p53 by interacting with *MDM2* through alternative splicing, thus activating cell cycle checkpoints (15). In NF1-associated MPNSTs, approximately 63% of cases (n=64) exhibit biallelic inactivation of *CDKN2A*, which is associated with the development of “cell-in-cell” (CIC) structures within cells (16, 17). Previous research has shown that the loss of *CDKN2A* releases G1/S cell cycle checkpoints, disrupts E-cadherin distribution, and inhibits myosin light chain phosphorylation (MLC-p). This leads to cytoskeletal remodeling, CIC formation, increased invasiveness, and immune. Notably, in NF1-related MPNSTs, the loss of *CDKN2A* may act synergistically with epigenetic silencing and mechanical restructuring at the cellular level, enhancing tumor heterogeneity and drug resistance. The spatiotemporal regulation of these processes, including stress responses to the tumor microenvironment, epigenetic plasticity, co-evolution of mutations, and epigenetic modifications, remains poorly understood (17). Whole-genome sequencing uncovered separate gene clusters in NFs and MPNSTs. Activation of specific genes results in the deposition of trimethylated histone H3K27me3 at the *CDKN2A* promoter, promoting tumor invasiveness (18). Additionally, simultaneous loss of CDKN2A/p16(INK4a) and p53 not only disrupts G1/S checkpoint control but also indirectly triggers the mTOR pathway and enhances RAS signaling cascades. However, further research is needed to fully understand the regulatory networks governing these interactions (18, 19).

To enhance the development of more effective therapies, a multidisciplinary collaboration involving pathology, hematology, and radiation oncology is essential. The current approach includes continuous maintenance therapy using oral MEK inhibitors, such as tramatinib, coupled with regular monitoring through ultrasound and MRI follow-ups scheduled every three months. A year and a half after diagnosis, the tumor size showed no significant change, and no metastases were detected in the lungs or other organs (clinical observation).

Recent advances in the diagnosis of NF1-associated peripheral nerve sheath tumors have significantly improved our understanding. Integrating histopathology and molecular features has become crucial for enhancing diagnostic accuracy and classification. The 2017 consensus established histological criteria distinguishing neurofibroma-like tumors (ANNUBP) from MPNST. However, relying solely on morphology has become insufficient due to advancements in diagnostic techniques, making molecular data essential. Traditional histopathology has limitations, particularly in identifying tumors with malignant potential or atypical features, which can impair diagnostic precision. Consequently, experts advocate a combined histological and molecular diagnostic approach to improve reliability and predict tumor behavior. Notably, Lucas CG (20) proposed updated classification guidelines emphasizing molecular markers, such as the biallelic inactivation of *CDKN2A/B*, which serve as key diagnostic adjuncts for ANNUBP, even when morphology is inconclusive. In addition to histology, loss-of-function mutations in genes such as *SUZ12*, *EED*, and *TP53*, as well as marked aneuploidy, are valuable molecular indicators of malignancy. Moreover, Lucas CG recommended terminology revision, replacing “low-grade MPNST” with “ANNUBP with proliferative increase” to more accurately reflect biological behavior and avoid potential misinterpretation associated with the term “malignant,” thereby offering a clearer understanding of tumor risk and characteristics.

A precise clinical diagnosis is crucial for customizing management plans. The integration of molecular analyses aids in identifying tumors at risk of malignant progression, enabling targeted interventions to prevent unnecessary treatment and ensure timely therapy. To utilize these diagnostic advancements, it is essential to maintain sampling quality, which involves ensuring adequate tissue collection during biopsy and systematically integrating histological and molecular findings into pathology reports. This process supports more precise and comprehensive clinical decision-making.

## 4 Conclusion

Overall, the diagnosis and treatment of NF1-associated MPNST emphasize the critical role of complete surgical resection, the potential of combination therapies, and the value of molecular profiling in guiding both treatment and research, particularly in sarcoma subtypes with ambiguous pathology or significant therapeutic heterogeneity. Future studies should expand on more comprehensive functional investigations to further unravel epigenetic regulatory mechanisms and validate potential targeted therapeutic strategies.

## Data Availability

The original contributions presented in the study are included in the article/supplementary material. Further inquiries can be directed to the corresponding author.
